# Targeting Melanoma with Cancer-Killing Viruses

**DOI:** 10.2174/1874357901711010028

**Published:** 2017-03-31

**Authors:** Tiantian Zhang, Yogesh R. Suryawanshi, Helene M. Woyczesczyk, Karim Essani

**Affiliations:** Laboratory of Virology, Department of Biological Sciences, Western Michigan University, Kalamazoo, MI, 49008, U.S.A

**Keywords:** Melanoma, Biomarkers, Oncolytic virotherapy, Tumor targeting, Tumor microenvironment, Immune enhancement, Immunotherapy, Combinational therapy

## Abstract

Melanoma is the deadliest skin cancer with ever-increasing incidence. Despite the development in diagnostics and therapies, metastatic melanoma is still associated with significant morbidity and mortality. Oncolytic viruses (OVs) represent a class of novel therapeutic agents for cancer by possessing two closely related properties for tumor reduction: virus-induced lysis of tumor cells and induction of host anti-tumor immune responses. A variety of viruses, either in “natural” or in genetically modified forms, have exhibited a remarkable therapeutic efficacy in regressing melanoma in experimental and/or clinical studies. This review provides a comprehensive summary of the molecular and cellular mechanisms of action of these viruses, which involve manipulating and targeting the abnormalities of melanoma, and can be categorized as enhancing viral tropism, targeting the tumor microenvironment and increasing the innate and adaptive antitumor responses. Additionally, this review describes the “biomarkers” and deregulated pathways of melanoma that are responsible for melanoma initiation, progression and metastasis. Advances in understanding these abnormalities of melanoma have resulted in effective targeted and immuno-therapies, and could potentially be applied for engineering OVs with enhanced oncolytic activity in future.

## INTRODUCTION

Although melanoma accounts for only 3-5% of all skin cancers, it is responsible for nearly 80% of all skin-cancer related deaths [1-3]. Like most other types of cancer, melanoma arises from a combination of genetic and epigenetic abnormalities. The genetic mutations such as over-activation of BRAF and Ras, which are found in respectively around 50% and 20% of metastatic melanomas, favor melanoma cell over-proliferation and tumor progression [4]. In addition, the innate and adaptive immune suppression further contributes to melanoma survival and metastasis, such as the loss of the expression of tumor specific antigens and tumor associated antigens (TAAs), the lack of co-stimulatory signals for T cell activation and the inefficient presentation of antigen to antigen presenting cells (APCs). Conventional cytotoxic therapies are not always effective for melanoma [5]. For example, the application of dacarbazine or vermurafenib resulted in less than 20% response rate in patients with malignant melanoma [6]. Although interleukin (IL)-2 seems beneficial to a selected group of patients, it is associated with significant toxicity [7].

Oncolytic viruses (OVs) emerge as a promising approach for melanoma therapy by possessing two closely-related properties. These include OVs’ ability to preferentially target and lyse the tumor cells and their capability to enhance antitumor immune responses [8]. While the antiviral mechanism exists in normal cells, the malignantly activated or abnormally regulated pathways in tumor cells often favor OVs’ infection and replication, therefore generating natural tumor selectivity for some viruses. For example, melanoma cells over-expressing Ras and harboring defective interferon (IFN)-signaling pathways are effectively infected by reovirus and vesicular stomatitis virus (VSV) [9]. In addition, the abnormal characteristics of tumors can be manipulated for generating or increasing virus oncoselectivity. For example, the promoters of tyrosinase or survivin genes that are over-expressed in melanoma, have been incorporated into virus genomes and used for the oncospecific targeting of engineered OVs [10, 11]. Further, OVs have been engineered to express immunostimulatory or immunomodulatory genes to increase their immumoreactivity and antiproliferative efficacy. Genes of cytokines such as granulocyte monocyte colony-stimulating factor (GM-CSF), IL-2, IL-12, IL-18 and IFN-γ have been incorporated into OVs [12, 13]. Among these cytokines, GM-CSF has been the most widely engineered into OVs to recruit immune cells, enhancing the OVs’ clinical benefit. Talimogene laherparepvec (T-vec) and JX-594, the two most advanced OVs approved for treating melanoma and head and neck cancer respectively, are modified herpes simplex virus (HSV) and vaccinia virus (VV) both expressing human GM-CSF [14]. By causing tumor lysis and immune cells recruitment, OVs armed with immunostimulatory genes exhibit higher efficacy in regressing tumors than immunostimulatory gene products alone. For example, the objective response rate was 26% including the complete response in 11% of patients with treatment of T-vec; while an objective response rate of 6% with 1% complete response rate was observed with treatment of GM-CSF [15, 16].

Here, we provide a review about the oncolytic virotherapy focused on melanoma treatment, emphasizing the successful strategies used to attain increased tumor specificity, oncolysis and immunoreactivity. This review also describes the promising strategies with challenges, which lead the way for future modifications and usage of OVs, and is divided into three sections. The first section reviews biomarkers including the established, prognostic and immunotherapeutic biomarkers. The biomarkers are not only applicable for diagnostic purposes, but also significantly useful in OV genetic modifications for targeting melanoma cells. The second section reviews the successful strategies of engineering OVs for melanoma regression, which can be broadly divided into four categories: (1) modification of virus tropism to the tumor cells by transductional and transcriptional targeting or cancer signaling pathway targeting; (2) triggering vascular-disrupting effects and modifying the extracellular matrix (ECM) components for virus spread; (3) viral enhancement of innate and adaptive antitumor immune responses; and (4) combinational therapies. The subsequent section focuses on the prospective useful strategies along with the challenges for optimizing the clinical efficiency of OVs.

## 1 BIOMARKERS OF MELANOMA

In recent years, the development of large-scale sequencing approaches has uncovered the complex molecular and cellular abnormalities leading to the tumorigenesis of melanoma. These discoveries have shown that melanoma is a genetically heterogeneous disorder with many distinctive molecular and cellular defects, which strongly suggest the individualization of diagnosis and treatment. American Joint Committee on Cancer (AJCC) melanoma clarifies TNM staging (T: primary tumor; N: regional lymph nodes; M: metastasis) and provides well-established hallmarks for melanoma progression such as tumor thickness, ulceration, mitotic rate and lactate dehydrogenase (LDH) [17]. Although these criteria correlate with the prognostic information, they are still unable to identify the prognosis of specific individuals and sometimes provide unreliable results. For example, some patients with thick tumors have excellent prognosis, and those with thin tumors result in poor outcome [18]. Therefore, addition of the measurable diagnostic indicators of melanoma, referred as biomarkers, would be likely to determine more accurately the prognosis. Also, biomarkers are able to determine which kind of treatment the patients would benefit from. Therefore, detection of these biomarkers would indicate the therapies needed for better outcomes. For example, the detection of BRAF mutation and administration of BRAF V600E kinase inhibitors such as dabrafenib significantly improve the clinical situation of the patients [6, 19]. In this section, we aim to briefly review the established biomarkers and biomarkers of interest in the last few years. These biomarkers not only identify high-risk melanoma patients, but also provide potential therapeutic targets for individualized treatment.

### 1.1 Established Biomarkers

#### 1.1.1 Thickness Measurement

Alexander Breslow [20] first reported the importance of using thickness and cross-sectional areas in reflecting the tumor burden. The prognostic significance of thickness measurement was also confirmed in the latest AJCC melanoma staging criteria. While 10-year survival rate is approximately 92% in patients with ≤ 1.00 mm thick melanoma, it drops to 50% in those with > 4mm thickness [17].

#### 1.1.2 Melanoma Ulceration

 The presence of tumor ulceration has been shown to adversely affect the disease outcome [17]. The ulceration caused by tumor eroding epidermis modifies the local environment and favors the expansion of the tumor burden [21]. Recent studies have shown that treatment of IFN-α significantly increase the survival rate in the patients with ulcerated melanoma in comparison to those with non-ulcerated melanoma, suggesting that ulceration might lead to decreased production of endogenous IFN [22, 23].

#### 1.1.3 Mitotic Activity

 DNA replication involves the activation of replication origin firing (ROF) genes and securin which separates sister chromatids [24]. Melanoma in patients with poor prognosis is often characterized with over-activation of ROF and expression of securin gene [24, 25]. Increased mitotic rate represents higher metabolic activity of tumor cells and therefore the increased tumor proliferation and metastasis.

### 1.2 Prognostic Markers

Among serological biomarkers, LDH remains to date the strongest prognostic markers in melanoma [26]. LDH is less sensitive in the early stage. However, it is indicative of the tumor growth and malignancy progression [27, 28]. It is still noteworthy that LDH, which occurs also in infection, inflammation and hemolysis, might lead to false positive readouts. S100 serves as diagnostic and prognostic marker for melanoma, as its expression is much higher in the malignant melanoma than in the normal tissues [29, 30]. It has been found that elevated level of S100, in particular S100B, is related to the poor prognosis and low survival rate [31, 32]. Vascular endothelial growth factor (VEGF) and melanoma-inhibitory activity (MIA) protein are involved in tumor-associated angiogenesis and melanoma cell interaction with the ECM respectively. Studies have shown that elevated levels of VEGF and MIA are associated with more advanced disease and poor survival rates [33, 34]. Other serum biomarkers for melanoma include tyrosinase [35], osteopontin [36], IL-8 [37] *etc*.

In addition to the serum biomarkers, molecules over-expressed in the tumor tissues also function as an effective indicator of the melanoma progression and metastasis. For example, matrix metalloproteinases (MMP), which proteolytically degrade ECM proteins, have been shown to promote melanoma cell migration and tumor progression. The MMP-1/3 positive melanoma was shown to correlate with higher malignancy and decreased overall survival [38]. Cell adhesion molecules (CAMs) such as the carcinoembryonic antigen-related cell adhesion molecule-1 and melanoma-associated antigen (MAA), which play an essential role in cell adhesion, show abnormal expression in melanoma and participate in melanoma migration and metastasis [39, 40]. Other tissue-specific biomarkers such as cyclooxygenase-2 (Cox-2) and galectin-3 have also been shown to associate with melanoma tumor size and malignancy and overall patient survival.

### 1.3 Genetic Biomarkers

About half of the melanomas harbor the mutation of BRAF gene (BRAF^V600E^, and possibly BRAF^V600K^), which results in constant activation of BRAF kinase [6]. This likely induces the promotion of Ras-Raf-MEK-ERK signaling pathway and therefore the proliferation of melanoma. Like BRAF mutation, *N-Ras* gene mutation is also considered as the “driver mutation” and found in approximately 15-20% metastatic melanoma patients [4]. BRAF and N-Ras mutations have been shown to link to the metastasis and poor outcome [41]. With lower frequencies, KIT mutations have been observed in mucosal and acral melanomas and melanomas with sun-damaged skin [42]. Cutaneous melanomas are characterized with mutations of BRAF, N-Ras, MEK, NF1 and KIT, while most ocular melanomas harbor the mutations of GNAQ and GNA11 [43]. In order to discriminate Spitz nevi and Spitzoid melanomas, biomarkers such as CDKN2A, RREB1, MYC and CCND1 have been utilized [44].

### 1.4 Biomarkers For Immunotherapy

Cytotoxic T-lymphocyte antigen-4 (CTLA-4), a protein receptor found on the surface of some T cells, functions as an immune checkpoint. By binding to the B7 on the APCs, it transmits an inhibitory signal to T cell activation, which confers vulnerability to the tumor invasion [45]. Ipilimumab (FDA approved for melanoma therapy in 2011), the human monoclonal immunoglobulin G1 against CTLA-4, has been shown to expand anti-tumor T cell activity and inhibit immune tolerance, therefore significantly enhancing the melanoma patients overall survival rate [46, 47]. Another anti CTLA-4 monoclonal antibody is tremelimumab, which is still undergoing human trials [48].

Programmed death receptor-1 (PD-1) is a protein expressed on the cell surface of T cells. By binding to the ligands PD-L1 and PD-L2 expressed on tumor and stromal cells, it transmits the signal in down-regulating T cell activation and promoting self-tolerance [49]. As PD-1 is also expressed on B cells and macrophages, it is likely more potent compared to CTLA-4 in negatively inhibiting immune responses [50]. PD-1 inhibitors, which block PD-1 and activate immune responses, have achieved varying success in different cancers including melanoma. Nivolumab, lambrolizumab and pembrolizumab, which are anti-PD-1 antibodies, have been approved by FDA for melanoma therapy [51, 52].

## 2 MELANOMA THERAPIES AND EMERGENCE OF ONCOLYTIC VIROTHERAPY

The traditional targeted therapies for melanoma include BRAF inhibitors (vemurafenib and dabrafenib), MEK inhibitors (trametinib and cobimetinib), tyrosine kinase inhibitors (imatinib) and angiogenesis inhibitors (aflibercept and bevacizumab) [6, 19, 53-60]. The melanoma immunotherapies include the cytokine therapies consisting of IFN-α and IL-2 [61-63] and inhibitors to CTLA-4 (ipilimumab) and PD-1 (nivolumab, lambrolizumab and pembrolizumab) [47, 51, 52].

OVs, with the initial clinical testing of the concept in 1950s [64], have achieved fast development over the past decade. With the ability of self-replicating in tumor cells and carrying additional genes for immune-stimulatory products, OVs have shown tremendous potential at being better anticancer therapeutics than the conventional drugs. It is encouraging that T-vec, a modified HSV strain expressing human GM-CSF, has been approved by FDA for melanoma therapy in 2015 [65]. The other most advanced oncolytic virotherapy used clinically is the JX-594, which has been approved in China for head and neck cancer therapy [66]. It is the oncolytic VV engineered by addition of GM-CSF gene and deletion of viral thymidine kinase gene. The expression of GM-CSF induces the recruitment and activation of APCs and therefore the tumor-specific T cell responses. Other viruses which are undergoing clinical tests for melanoma therapy include reovirus and coxsackievirus [9, 67].

### 2.1 Transductional Targeting

One of the major challenges of engineering OVs is that the natural tropism of viruses does not match the therapeutic need. For example, the administration of adenovirus systemically, among which the majority is sequestered by hepatic macrophages and hepatocyte transduction, results in adenovirus-mediated liver toxicity [68]. Therefore, approaches that retarget the viruses are needed to avoid the toxic side effects and facilitate the efficient infection to the targeted tumor cells. By modifying the viral coat proteins, which is referred to as transductional targeting, the viruses are engineered to infect the tumor cells that they do not infect naturally [69]. For example, the modification of hypervariable loop 5 in the capsid protein hexon in adenovirus prevents infection of hepatocytes and expand the anticancer therapeutic window [70].

Pseudotyping, which is the first approach of transductional targeting, is to replace viral attachment proteins of one virus with the tropism determinant ligands of other viruses whose tropism is against the targeted tumor cells. Exciting results have been observed by using oncoretroviral vector pseudotyped with a modified chimeric Sindbis virus envelop glycoproteins and conjugated with antibody specific for P-glycoprotein, for targeting metastatic melanoma [71].

While pseudotyping is limited by the viral attachment proteins and the receptors exclusively present on the target cells, the other approach of transductional targeting by using adaptors, the molecules with one end binding the viral attachment protein and the other binding the target cells’ receptors, possesses more flexibility without changing the structure of viral vectors [72]. For example, the fusing of ectodomain of coxsackie virus and adenovirus receptor (CAR) and a single chain antibody against carcinoembryonic antigen allowed the adenovirus to target on tumors and ablated the liver tropism [73]. Avidin, Biotin and monoclonal antibodies are also used in adaptor systems. For example, a biotin-acceptor peptide has been incorporated into the adenovirus fiber capsid protein and biotinylated and coupled to an avidin-binding ligand [74]. MMP and MAA, as the biomarkers of melanoma, play a critical role in melanoma progression and metastasis. Efficient targeting of retrovirus to melanoma cells has been done by fusing a single chain antibody recognizing the high molecular weight MAA (HMWMAA) followed by a proline-rich linker and a MMP-2 cleavage site, to the amino terminus of murine leukemia virus (MLV-A) envelop glycoprotein [75, 76]. Following the attachment with HMWMAA, MMP cleaves the MMP-2 cleavage site and allows the infection of MLV. Another example combining the avidin/biotin and monoclonal antibody is by first biotinylating VV to which the avidin is subsequently added and then coating the avidin bridge with biotinylated antibodies. The antibodies are against the cell surface makers of target cells [77].

### 2.2 Transcriptional Targeting

The transcriptional regions consisting of promoters and enhancers regulate the kinetics and levels of mRNA production, and activators recruit RNA polymerase II for initiating the transcription of mRNA. Cancer-specific promoters can be activated in the presence of a subset of activators existing in cancerous cells but not in normal cells [78]. Therefore, transcriptional targeting in which part of the viral genome is placed under the control of cancer-specific promoter serves as a feasible way for generating tumor selectivity.

Tissue targeted virotherapy has been investigated in a variety of cancers including melanoma. Promoters of genes over-expressed in melanoma have been chosen and utilized, among which most are the above mentioned melanoma biomarkers’ promoters such as Cox-2, C-X-C chemokine receptor type 4 (CXCR-4), tyrosinase and survivin promoters [10, 78-80]. Cox-2 is closely associated with the progression and metastasis of melanoma, whereas benign nevi and tissues are Cox-2 negative [81]. CXCR-4 over-expressed on human melanoma cells has also been shown to play a role in melanoma progression [82]. Incorporation of Cox-2 and CXCR-4 promoters in adenovirus genome has been shown to significantly enhance the virus-mediated gene expression in the malignant melanoma instead of non-malignant primary melanocytes [79, 80]. Tyrosinase, which plays an essential role in melanin synthesis and melanocyte differentiation, has been used as a melanoma biomarker for disease detection purposes [83]. It has been demonstrated that incorporation of tyrosinase promoter to drive the expression of the viral genes of adenovirus leads to an obvious melanoma-selective viral cytotoxicity [84]. Survivin is expressed on a variety of tumors but undetectable in terminally differentiated tissues. It is associated with tumor progression and resistance to chemo- and radio-therapies, which is likely due to its ability of modulating apoptosis pathways and inhibiting apoptotic enzymes such as caspase-3 and caspase-7 [11, 85]. Lu *et al.* have reported that compared with Cox-2 and CXCR-4 promoters, the activity of survivin promoter is higher in melanoma cells and lower in normal tissues such as livers and epithelial melanocytes [79]. A continued search for a better cancer-specificity is warranted, as higher expression in tumor cells and lower expression in normal tissues for transgenes in viral vectors is always desirable.

### 2.3 Targeting Cancer Signaling Pathways

Mutations in *ras* gene, which lead to the constant activation of Ras and therefore cell over-proliferation and tumorigenesis, have been reported in 30% of all cancers [86]. The incidence of *ras* mutations in melanomas is around 20% and the majority is *N-ras* mutations [87]. It has been demonstrated that reovirus and VSV, in unaltered form, replicate preferentially and effectively in the Ras-mutated cancer cells and therefore serve as natural OVs [88, 89]. It has been shown that reovirus efficiently infects and kills melanoma cells and regresses the xenografted melanoma tumors. In addition, Ras signaling pathway has been shown to be involved in reovirus-induced apoptosis in human melanoma cells [9]. Similarly, Noser *et al.* [89] have reported that Ras-overexpressing cancer cells are effectively killed by VSV and the oncolytic properties of VSV are dependent on the activation of Ras/Raf/MEK/ERK signaling pathway. It is noteworthy that the activation of Ras pathway is usually coupled with the down-regulation of IFN-induced antiviral responses such as the induction of protein kinase PKR, which further favors the virus replication in cancer cells.

Dysfunction of IFN signaling pathway has been reported in different cancers such as melanoma and breast cancer. Impaired IFN pathway aids tumor progression and immune escape, as IFN signaling is critical for the proper activation of key immune cells such as T, B and natural killer (NK) cells [90]. It has been reported that several melanomas are deficient in the key components of IFN signaling pathway such as STAT1 and STAT2 [91]. One example of enhancing the viral oncoselectivity via targeting the impaired IFN pathway in tumors involves in the nonstructural protein NS1 of influenza virus which has been demonstrated to block the function of PKR induced by IFN [92]. Influenza virus with NS1 deleted has been shown to selectively infect and lyse the melanoma cells that are PKR-deficient and regress the melanoma tumors *in vivo*, while failing to replicate in normal cells with intact PKR-mediated antiviral responses [93]. It is worthwhile to further investigate the application of viruses with the IFN or PKR viral inhibitor deletion for melanoma virotherapies, as IFN/PKR inhibitors are expressed by a variety of viruses such as VV and retrovirus [93].

Deficiency in apoptosis is a critical contributor to the therapeutic resistance of melanoma. Two pathways, the intrinsic and extrinsic pathways lead to the apoptosis. While Bcl-2 family proteins such as Bax and Bak and the induction of apoptosome and caspases are involved in the intrinsic pathway, the extrinsic pathway features the death ligands of tumor necrosis factor (TNF)-α protein family including TNF-α, Fas ligand (CD95L) and TNF-related apoptosis-inducing ligand (TRAIL) [94]. The application of tyrosinase promoter controlled CD95L has been shown to selectively induce apoptosis in melanoma cells while sparing the non-melanoma cells [95]. Viruses such as Newcastle disease virus (NDV) have been demonstrated to possess the ability of naturally inducing apoptosis in various cancer cells [96]. A wide array of viral proteins which have been shown to induce apoptosis include: NS3 protein of bovine viral diarrhea virus, viral protein R of human immunodeficiency virus, hemagglutinin-neuraminidase of NDV and EIA of adenovirus [96-99]. It is appropriate to incorporate these apoptosis-inducing proteins into the OVs for expression. In addition, the over-expression of anti-apoptotic proteins is a critical factor for melanoma’s resistance to the chemotherapy. Therefore, it is feasible to increase the viral oncolysis by co-administrating with inhibitors of the anti-apoptotic proteins, such as BCL-2 inhibitors. It has been reported that the co-treatment of BCL-2 inhibitor improves the viral oncolysis of VSV in the tumors which are resistant to the treatment of virus alone [100].

### 2.4 Targeting Tumor Micro-Environment

The components in the environment where tumors exist, including the blood vessels, fibroblasts, immune cells and elements of ECM, are collectively termed as “tumor microenvironment”. The interaction between tumor cells and tumor microenvironment is critical in tumor initiation, progression and invasiveness. Also, tumor microenvironment affects the delivery and spread of the therapies such as OVs in tumors. Therefore, in this section, we will review the strategies used for increasing the efficacy of OVs and combating the melanoma progression via targeting the melanoma tumor microenvironment.

#### 2.4.1 Targeting Tumor Vasculature

The formation of neovasculature is essential for the supply of nutrients and oxygen and melanoma cell dissemination. Therefore, the angiogenesis and lymphangiogenesis are critical for melanoma progression and metastasis [101]. Angiogenesis of melanoma is stimulated by the overproduction of growth factors such as VEGF, fibroblast growth factor (FGF), platelet-derived growth factor (PDGF), and transforming growth factor (TGF). The rapidly increased vessel density and compression of the melanoma cells lead to the hypoxic and acidic environment throughout the tumor, which in turn induces the enhanced production of VEGF, FGF and TGF [102]. In this regard, drugs inhibiting angiogenesis such as anti-VEGF antibody (bevacizumab and aflibercept) have been designed and utilized.

Targeting tumor angiogenesis has been used as an effective strategy for attaining increased tumor regression of OVs. It has been reported that the mutant VV engineered to express GLAF-1 which is the single-chain antibody targeting human VEGF, exhibited significantly increased tumor regression in human tumor xenografts compared with the parental virus [103]. The HSV armed with transgene of platelet factor 4 which is an antiangiogenic agent has been shown to be more efficacious in inhibiting the tumor growth than the parental HSV [104]. In addition, the incorporation of VEGF-targeted short hairpin RNA in the adenovirus results in greater tumor inhibition [105]. Cytokines such as IL-12 and IL-18 have also been shown to reduce tumor angiogenesis [106]. Adenovirus expressing IL-18 has exhibited greater apoptosis-induction and antitumor effect in xenografted human melanoma through inhibiting angiogenesis [107].

#### 2.4.2 Targeting ECM

Effective oncolysis requires the virus entry and the subsequent replication and spread in the tumor environment. Therefore, a variety of strategies have been utilized to modify the physical aspects of tumor microenvironment to attain the optimal virus dispersal and replication.

Consistent with this idea, transgenes of enzymes degrading ECM components such as collagenase, hyaluronidase and MMP have been incorporated into OV modifications. The treatment of collagenase prior to the injection of adenovirus has been shown to enhance the virus spread [108]. Similarly, the expression of hyaluronidase by the mutant adenovirus resulted in improved spread of the virus within the tumor and increased antitumor efficacy [109]. MMPs, in particular, MMP-1 and MMP-8 involve in degrading the triple-helical collagen in ECM. As melanoma biomarkers, MMPs correlate closely with melanoma metastasis and decreased overall survival rate [38]. Intratumoral injection of mutant adenovirus constructed to express MMP-8 led to significantly reduced collagen expression and increased tumor eradication [110]. In addition, engineered OVs expressing relaxin and decorin, which are known as the ECM modulating proteins, have yielded enhanced virus penetration, persistence and spread compared to the control virus in melanoma [102, 111, 112].

### 2.5 Targeting Immune Response Enhancement

Oncolytic virotherapy may represent a unique combination of viral tumor lysis and the activation of innate and adaptive immune responses. Targeted infection of tumor cells causes the release of TAAs and localized inflammation, which subsequently induces the tumor recognition of host immune system [113]. Therefore, in this section, we will review the strategies for enhancing immune recognition of cancer specific neo-antigens and antitumor immune responses.

#### 2.5.1 Arming OVs with TAAs

TAAs are the antigenic substances produced in tumor cells that trigger the immune responses towards the tumor. Certain tumor antigens, such as tyrosinase and melanoma–associated antigen (MAGE) in melanoma, are therefore used as the biomarkers for diagnostic test or the development of cancer therapies [114].

The combination of virus-mediated tumor destruction and enhanced immune recognition of TAAs has been studied and exhibited encouraging results. Use of VSV as the vector expressing TAAs from the cDNA library of melanoma successfully induced IL-17 recall response of tumor specific CD4^+^ T cells and eradicated melanoma tumors [115]. Also, VSV expressing epitope library of human prostate cancer induced rejection of established prostate tumors [116]. These results warrant further studies by using OVs expressing cDNA of multiple selective TAAs for activating a wide repertoire of APC and T cell responses.

#### 2.5.2 Arming OVs with immuno-stimulatory or immune-modulatory genes

Tumors have been evolving to escape the antitumor immune response and develop the therapy-resistance. The infection and lysis of tumor cells by OVs triggers the localized release of inflammatory cytokines which subsequently attract the immune cells for tumor elimination [115]. However, it is questionable if the cytokines are sufficient for eliciting tumor-reduction immune responses. Therefore, it would be desirable to have OVs expressing cytokines effective in inducing antitumor immune responses.

The two most advanced OVs, T-vec and JX-594 are respectively the modified HSV and VV, both expressing human GM-CSF which recruits dendritic cells and NK cells and induces tumor-specific cytotoxic T lymphocytes responses [117]. T-vec shows significant improvement of durable response rate and overall survival rate of patients with advance melanoma [15].

The induction of anti-tumor T cell responses requires two signals: (1) the interaction between the major histocompatibility complex protein presenting tumor antigens and T cell receptors, and (2) the costimulatory molecules on APCs or tumor cells [118]. The costimulatory signals come from the binding of B7 on activated APCs to CD28 or CTLA-4 on T cells and the binding of CD40 on APCs to CD40L on T cells. While interaction of B7 and CD28 promotes T cell activation and survival, the binding of B7 and CTLA-4 transmits inhibitory signal to the activated T cells. The binding of CD40L and CD40 triggers T lymphocyte expansion and increases IL-12 release which is critical for the engagement of CTL for the antitumor immune response [119, 120]. Adenovirus, VSV and HSV engineered to express soluble B7 or CD40L exhibited more effective melanoma regression and significantly enhanced CD4^+^ and CD8^+^ T-cell infiltration [121-123]. Similarly, potent antitumor activity along with increased T cell activity have been observed with the administration of adenovirus and VSV coding a fully monoclonal antibody specific for CTLA-4 [124, 125]. Binding of PD-L1 or PD-L2 on tumor cells to PD-1 on T cells inhibits antitumor T cell response. Combination of Anti-PD-1 blockade with virotherapy has been shown to improve the virus’ antitumor activity and result in improved therapeutic outcome [126].

A variety of cytokines have been incorporated into OVs (Table 1) to activate various lymphocytes and attain enhanced antitumor immune response. For example, IL-2, the T cell growth factor, not only promotes T cell activation and proliferation, but also activates NK cells and increases NK cells’ lysing ability. IL-2 has been engineered into viruses such as NDV and HSV, intended to stimulate the activation of T and NK cells [127-129]. Other cytokines engineered into OVs for promoting the antitumor activity through enhancing immune cell activation include IL-12, IL-15, IL-18, CCL-3 and IFN-γ *etc* [12, 13, 123, 130-134]. The expression of these cytokines results in overall increase of therapeutic effect, despite the effect that some cytokines such as GM-CSF and IFN-γ cause reduced virus replication and early clearance.

### 2.6 Combination of OVs and Other Therapies

In the past decade, there has been a rapid development of viruses with oncolytic activity as the clinical or preclinical anticancer therapeutics. More and more studies have shown that rather than using OVs as tumor therapy alone, it would be likely to realize OVs’ full therapeutic potential by combining with other treatment strategies such as radiotherapy, chemotherapy or immunotherapy.

#### 2.6.1 Oncolysis with Radiotherapy

The combination of OVs and radiotherapy has resulted in synergistic therapeutic effect, which significantly inhibits the tumor growth relative to either of the individual therapies [135]. It has been reported that radiation exposure significantly increased the virus replication in a variety of cancer cell lines *in vitro* and *vivo* [136-140].

The prospective synergistic efficacy by combining OVs and radiotherapy in melanoma is due to four aspects. First, OVs and radiation individually shows therapeutic efficacy in melanoma treatment. Second, radiation may reduce the high pressure of tumor cells which serves as an obstacle for virus replication and spread *in vivo*. Third, Ras mutation is one of the driver mutations for melanoma and is associated with radio-resistance [141-143]. However, reovirus, VSV and HSV have been demonstrated to preferentially target the Ras mutated melanoma cells and induce apoptosis [88, 89]. Therefore, OVs which kill the radiation-resistant melanoma cells will exert a complementary therapeutic effect to radiotherapy. Fourth, infection of the melanoma cells induces the release of localized inflammatory cytokines such as TNF-α and TRAIL, which have been shown to sensitize the cells to the effects of radiation [144-148]. The combination of reovirus and radiation has shown to increase the tumor growth delay of the melanoma xenografts in the treated animals, and significantly enhance the overall survival rate compared to the treatment with either of the individual therapies [149].

#### 2.6.2 Oncolysis with chemotherapy

Chemotherapies can be generally divided into several categories: (1) alkylating agents that bind covalently to DNA via their alkyl group and exert antitumor effect, such as cyclophosphamide (CPA), decarbazine, cisplatin and carboplatin; (2) anti-metabolites that inhibit DNA and RNA synthesis, such as fluorouracil; (3) anti-microtubule agents that interfere with the microtubule function and inhibit cell division, such as paclitaxel and docetaxel; (4) topoisomerase inhibitors that prevent DNA replication and translation, such as novobiocin and aclarubicin; and (5) cytotoxic antibiotics that interrupt cell division, such as mitomycin C and doxorubicin [150]. With the consideration that chemotherapy and OVs work with different mechanisms via different pathways, it is probable that the combination of these two therapies would exert synergistic therapeutic effectiveness.

The preclinical study of combinational therapy on malignant melanomas has demonstrated the difference between a variety of chemotherapies in enhancing the viral oncolysis. Pandha *et al.* [151] have reported that reovirus, in combination with cisplatin or paclitaxel, resulted in more pronounced tumor-killing effect in malignant melanoma than with other chemotherapeutic agents such as dacarbazine, gemcitabine and carboplatin, despite the fact that synergistic effect has been seen when reovirus was combined with all these chemotherapeutic agents. The combinational therapy of reovirus and cisplatin resulted in more remarkable retardation of tumor growth, in comparison to using cisplatin or reovirus alone. In addition, the intravenous administrations of combinational therapy of reovirus with paclitaxel, carboplatin, dacarbazine and gemcitabine are undergoing phase I or phase II trials [152].

It is critical to understand the interplay between the virus and the chemotherapies of choice which likely modify the humoral and adaptive immune responses. For example, CPA has been reported to ablate the neutralizing antibodies against the virus intratumorally administered, and it is necessary to maintain sufficient levels of neutralizing antibodies to prevent the virus to cause toxicities in systemic organs [153]. Therefore, it is critical to analyze the toxicity, carefully make chemotherapy dose decisions and supervise the real-time levels of neutralizing antibodies against virus.

#### 2.6.3 Oncolysis with Immunotherapy

The current immunotherapy for melanoma include cytokine agents (IL-2 and IFN-α) and antibodies against CTLA-4 and PD-1. Targeting CTLA-4 and PD-1/PD-L1 pathways via using anti-CTLA-4 and anti-PD-1/anti-PD-L1 has achieved more efficacies in regressing melanoma, as compared to other cancers such as renal cell carcinoma [154]. Combining two therapeutic agents with different mechanisms of action is highly desirable, due to the potentially enhanced efficacy than that of single drug alone. While in some instances the combinations lead to promising effectiveness, some do not. For example, a phase II trial in metastatic melanoma patients has shown that compared with ipilimumab (CTLA-4 blockade) monotherapy, the combination of ipilimumab and GM-CSF resulted in enhanced overall survival rate and tumor reduction with less toxicity [155]. However, a phase I study of cotreatment of ipilimumab and vemurafenib demonstrated remarkable increase of the liver toxicity than that of ipilimumab single treatment, which ultimately caused the early termination of the clinical trial of this combination [156].

In regard of these findings, cotreatment of immunotherapy and OVs provides another appealing option for enhancing the therapeutic effect for melanoma treatment. Rajani *et al.* [157] demonstrated that intravenous injection of anti-PD-1 and intertumoral injection of reovirus enhanced the survival of mice with melanoma xenografts compared to either therapy alone. Addition of anti-PD-1 not only reduced the T-reg activity and increased the cytotoxic T cell-mediated tumor reduction, but also significantly enhanced the NK cell activation mediated by the reovirus-infected tumor cells [157]. Similarly, it has been shown that compared with combination with anti-TGF or anti-IL-10R, combination with anti-CTLA-4 strikingly improves the therapeutic effect of VSV-mediated tumor regression by eliciting CD4^+^ and CD8^+^ T cells response [124]. IL-2 has been known as the T cells growth factor and is able to enhance the proliferation of T cells and activate NK cells and macrophages. It has been shown that OVs expressing IL-2 successfully increased the regulatory CD4^+^ and cytotoxic CD8^+^ T cells proliferation and memory T cells generation, resulting in effective reduction of tumors [128, 158]. In addition, Kottke *et al.* [159] have shown that systemic IL-2 causes the endothelial cell injury (vascular leak syndrome), which facilitates the efflux of circulating VSV to locate into melanoma tumors and increases the number of NK cells.

### FUTURE DIRECTIONS

A major part of the current standard therapeutic strategy for melanoma includes a range of targeted treatment entities based on specific gene mutations (BRAF inhibitors), pathways or enzymes (MEK inhibitors, tyrosine kinase inhibitors) and growth factors (VEGF inhibitors) [6, 19, 53-60]. Melanoma associated biomarkers like LDH, MIA, VEGF and MMP have shown a notable prognostic value where the level of these biomarkers has closely correlated with the advancement of the disease [33, 34, 38]. In the context of FDA approved therapies targeting melanoma biomarkers such as BRAF, MEK and VEGF, it is safe to say that biomarkers can not only serve as prognostic tools but are also useful in developing new therapies targeting melanoma. Targeted therapies fail to provide expected outcomes in many patients due to the genetic variability of melanoma, as melanoma tumors can be genetically heterogeneous and may or may not harbor mutations in target genes of therapeutic interest. Efficacy of treatment strategy for melanoma increases significantly when multiple targeted therapies are used in combination versus use of a single therapeutic agent, indicating the high level of dependence of targeted melanoma therapies on the genetic profile of the cancer. While nearly 50% of melanoma patients usually respond to BRAF inhibitors, the number of non-responding patients decreased by 40% when BRAF inhibitors were used along with MEK inhibitors as a combination therapy [6, 19, 55, 160]. In regard of this, immunotherapy (IFN-α and IL-2) and oncolytic virotherapy that are not heavily dependent on genetic heterogeneity of melanoma would circumvent limitations associated with targeted therapies that target specific gene mutations.

The OVs’ ability of causing tumor lysis, stimulating local inflammatory responses and serving as gene delivery vehicles, supports them as promising therapeutic agents for melanoma. While OVs are able to induce cell lysis and local inflammation that are not relying on genetic heterogeneity of melanoma, some viruses, in native forms, preferentially infect melanoma cells with aberrant genetic properties. Viruses such as reovirus, NDV and VSV have been shown to effectively kill the Ras-mutated tumor cells or lead to apoptosis, which make them as natural OVs for melanoma [88, 89, 96]. In addition, for those viruses without natural oncoselectivity, modifications targeting them to the genetic abnormalities of melanoma, often represented as biomarkers, have shown significant effectiveness in enhancing their oncolytic activity. For example, promoters of melanoma biomarkers such as Cox-2, CXCR-4, tyrosinase and survivin have been incorporated into the viral genomes, which result in significantly enhanced oncospecificity [10, 78-80]. A single chain antibody recognizing the HMWMAA, which is another melanoma biomarker, has been engineered into the virus for transductionally targeting melanoma cells [77]. Beyond the genetic and epigenetic alterations within the melanoma cells, the interaction between the melanoma cells and the microenvironment critically determines the transformation process of melanoma. Microenvironmental factors such as growth factors (VEGF, FGF, PDGF and TGF) and proteolytic enzymes (MMPs) involve in angiogenesis and tissue remodeling, highly promoting melanoma progression and metastasis. Modifications of OVs to target these microenvironmental factors have resulted in enhanced viral penetration and spread and melanoma regression [103, 110, 111].

OVs represent a class of novel agents as they can be engineered to harness the benefits of targeted therapy and immunotherapy, by inducing cancer cell death along with induction of a strong innate and adaptive immune response against cancer cells. The greatest success has been achieved with HSV (T-vec) and VV (JX-594) when developing OVs for cancer treatment. Some other viruses that are being studied in pre-clinical and clinical trials include HF10 (HSV), reovirus, NDV, VSV and coxsackie virus [115, 127, 152, 161, 162]. TAAs and immunostimulatory cytokines have been engineered into OVs, intended to attain enhanced immune recognition of tumor antigens and antitumor immune responses [115]. Using the viruses as the platforms for expressing immunostimulatory cytokines such as GM-CSF is appealing as it is likely to increase the local concentration of the gene products and minimize the side effects due to the systemic exposure [163]. While in some instances the expression of cytokines showed no inhibitive effect in virus replication, in some other cases the incorporation of cytokines such as IL-2, IFN and GM-CSF resulted in significantly decreased virus titer [126, 128, 164-166]. To cope with this issue, strategies have been designed to fuse the cytokine transgene to an exogenous cytokine-expressing regulator, which regulates the expression of the cytokine at specific time [167].

While some OVs are being studied for their efficacy as monotherapy, some are under investigation in combination with other therapies such as chemotherapy, radiotherapy, targeted therapy and more recently adoptive T cell therapy. T-vec is under investigation in several clinical studies where its efficacy is being assessed when combined with ipilimumab (CTLA4 inhibitor), and the results of a phase Ib/2 study of T-vec+ipilimumab suggest the enhanced synergistic efficacy with higher complete response and objective response rate [168]. Similar synergistic effects have also been observed when OVs were combined with anti-PD-1 and IL-2 [157]. Adoptive T cell therapy, with priming of T cells against tumor antigens followed by transfusion of the primed T cells in cancer patients, has shown consistent efficacy in clinical trials for melanoma [169]. Adoptive T cell therapy is also being investigated as combination therapy along with OVs. It has been shown that oncolytic adenovirus expressing IFN-γ combined with adoptive T cell therapy resulted in overall objective response of 38.5% in melanoma patients [170].

Although combination therapies have shown significant improvement and encouraging results, careful attention is still paid to the clinical trial designs, timing and dosing regimens, biosafety and biodistribution. For example, when combining the OVs and immunotherapy such as anti-PD-1, the timing of immunotherapy administration would be critical to avoid augmenting the anti-viral immune response or any earlier clearance of the virus. Rajani *et al.* [157] have demonstrated that administration of anti-PD-1 7 days after the intratumoral injection of virus is optimal to maximize the virus spread within the tumors. When combining OVs with chemotherapies such as CPA, CPA is able to eliminate the neutralizing antibodies against the virus and allow the virus to cause toxicities in systemic organs [153]. Therefore, it is essential to critically supervise the toxicity and the real-time levels of neutralizing antibodies against the virus, and carefully make dose-escalation decisions. For the combinational therapy of OVs and immunotherapy, it has been proposed to additionally combine with the systemic treatment such as T-reg depletion, to exert even higher synergistic efficacy [125]. However, one should be cautious to avoid autoimmune reactions when combining T cell enhancement with T-reg depletion, as immunotherapy such as anti-CTLA-4 treatment is already closely related to decreased number of myeloid derived suppressor cells [171].

Although it is reasonable to expect sufficient efficacy exerted by a one-shot curative agent, the immune “barriers” such as virus neutralization and sequestration make it more realistic to attain the optimal therapeutic effect with serial OVs for priming the antitumor immune responses. Strategies have been developed to prime the antitumor responses with one viral vector and boost with another, in order to minimize the anti-viral immune responses [172, 173].

### CONCLUSION

In summary, this review describes OVs and their mechanisms in melanoma tumor regression. We also discuss different combinational therapies, which overcome the limitations associated with each therapeutic agent such as chemo-, radio- and targeted- therapies. Nonetheless, the quest for identifying new melanoma biomarkers that can channelize efforts for developing newer targeted therapies for melanoma should be continued.

## Figures and Tables

**Table 1:** Melanoma virotherapy.

**Virus**		**Modifications**	**Experimental**	**Clinical trials**	**References**
**Herpes simplex virus**	T-vec	ICP34.5 deletion, US11deletion, GM-CSF insertion	+	+	14, 15, 16, 65, 117
	HF10	UL56 deletion	+	+	161
		IL-2 insertion	+	-	129
**Vaccinia virus**	JX-594	GM-CSF insertion, TK deletion	+	+	14, 66, 117
**Reovirus**	Reolysin	None	+	+	152
**Coxsackie virus**	Cavatak	None	+	+	162
**Adenovirus**		Tyrosinase promoter/enhancer insertion	+	-	84
		IL-18 insertion	+	-	107
		IL-12 and B7 insertion	+	-	123
		Decorin insertion	+	-	102
**Vesicular stomatitis virus**		Insertion of TAAs from melanoma cDNA library	+	-	115
		CD40L insertion	+	-	121
**Influenza virus**		NS1 deletion	+	-	93
**Newcastle disease virus**		IL-2 insertion	+	-	127, 128
**Measles virus**		Encoding anti-CTLA-4 and anti-PDL1	+	-	126
**Retrovirus**		Insertion of anti-HMWMAA	+	-	75, 76, 77

**Abbreviations:** CD, cluster of differentiation; CTLA, cytotoxic T-lymphocyte antigen; GM-CSF, granulocyte monocyte colony-stimulating factor; HMWMAA, high molecular weight melanoma-associated antigen; ICP, infected cell protein; IL, interleukin; NS, non-structural; PDL, programmed death receptor; TAA, tumor associated antigen; ligand; TK, thymidine kinase; US, unique short.

## ABBREVIATIONS

AJCC: = American Joint Committee on Cancer
APC: = antigen presenting cell
CAM: = cell adhesion molecule
CAR: = coxsackie virus and adenovirus receptor
CD: = cluster of differentiation
Cox: = cyclooxygenase
CPA: = cyclophosphamide
CTLA: = cytotoxic T-lymphocyte antigen
CXCR-4: = C-X-C chemokine receptor type 4
ECM: = extracellular matrix
FGF: = fibroblast growth factor
GM-CSF: = granulocyte monocyte colony-stimulating factor
HMWMAA: = high molecular weight melanoma-associated antigen
HSV: = herpes simplex virus
IFN: = interferon
IL: = interleukin
LDH: = lactate dehydrogenase
MAA/MAGE: = melanoma-associated antigen
MIA: = melanoma-inhibitory activity
MLV: = murine leukemia virus
MMP: = matrix metalloproteinase
NK: = natural killer
NDV: = Newcastle disease virus
OV: = oncolytic virus
PD: = programmed death receptor
PDGF: = platelet-derived growth factor
PK: = protein kinase
ROF: = replication origin firing
T-vec: = talimogene laherparepvec
TAA: = tumor associated antigen
TGF: = transforming growth factor
TNF: = tumor necrosis factor
TRAIL: = tumor necrosis factor-related apoptosis-inducing ligand
VEGF: = vascular endothelial growth factor
VSV: = vesicular stomatitis virus
VV: = vaccinia virus
